# Alzheimer's research after full approval of lecanemab: impetus and variety

**DOI:** 10.1590/1980-5764-DN-2023-0064

**Published:** 2024-01-05

**Authors:** Timothy Daly

**Affiliations:** 1 Bioethics Program, FLACSO Argentina, Tucumán, Buenos Aires, Argentina.; 2 Sorbonne Université, Science Norms Democracy, Paris, France.

Dear Editor,

The full US Food and Drug Administration approval on July 6^th^, 2023, of Eisai/Biogen's Leqembi (lecanemab) was a historic moment in research into Alzheimer's disease (AD), as it represents the first full approval of a biological treatment for AD in 20 years. This is crucial for the place of AD research in society, marked by decades of failure of clinical trials to meet their primary endpoints, and will hopefully attract more funding for research efforts. Because of this impetus, it is generally a good time to be an AD researcher.

However, it is vital that the optimism towards the dominant therapeutic strategy — the lowering of amyloid-β protein that along with tau defines the disease — does not hinder the promotion of therapeutic variety in AD research^1^. It is heartening that the most recently published update to the AD drug development pipeline shows significant heterogeneity of targets^2^. However, the pipeline shows only the end result of decades of work: the production of scientific knowledge on AD requires the work of thousands of researchers worldwide, and the most important resource within this knowledge-driven or “epistemic” community is credibility^3^. There has been long-standing belief in amyloid-lowering, popular among powerful researchers with the most credibility, to the extent that defenders of other theories have struggled to publish impactful research^4^. In other words, getting those non-amyloid targets into clinical trials has required decades of lobbying on the part of different AD researchers.

But why is theoretical and therapeutic variety in science important? The most important reason is that we still do not know the long-term worth of amyloid-lowering to AD patients. The current model of 12- to 18-month trials that led to support for lecanemab and similar antibodies represents a small part of the disease spectrum, and there is community-wide disagreement about the effect of the small benefit beyond the trial window. Planche and Villain^5^ argue that there are three anti-Aβ scenarios in AD: a symptomatic effect (no slowing), an enduring effect (early slowing), and an increasing effect (sustained slowing across disease stages). They consider that current trials cannot be used to determine which of these scenarios is correct and argue for innovative trial methodology to settle this question. Longer trials could also be useful^6^.

In this optimistic period, it is vital that belief about amyloid-lowering does not mislead the community, for both short- and longer-term reasons. As for short-term concerns, anti-Aβ antibodies are costly, frequent infusions that increase the risk of brain injuries in the form of bleeding and swelling, which can have fatal consequences^7^. As for mid- to longer-term concerns, beliefs about the value of anti-Aβ strategies to patients should be settled through aforementioned innovative trials^5^ and/or longer ones^6^. Finally, in the long term, drug development for AD should be funded according to a fair-share principle that tests the value of therapeutic targets in AD in ways that respect both scientific pluralism and therapeutic plausibility^8^.

In conclusion, by leveraging impetus and variety, the AD community can maximize its chances to develop clinically-meaningful treatments for current and future patients.

## References

[1] Daly T (2023). Lecanemab: turning point, or status quo? An ethics perspective. Brain.

[2] Cummings J, Zhou Y, Lee G, Zhong K, Fonseca J, Cheng F (2023). Alzheimer's disease drug development pipeline: 2023. Alzheimers Dement (N Y).

[3] Irzik G, Kurtulmus F (2021). Distributive Epistemic Justice in Science. Br J Phil Sci.

[4] Daly T, Houot M, Barberousse A, Petit A, Epelbaum S (2021). A Proposal to Make Biomedical Research into Alzheimer's Disease More Democratic Following an International Survey with Researchers. J Alzheimers Dis Rep.

[5] Planche V, Villain N (2023). Advocating for Demonstration of Disease Modification-Have We Been Approaching Clinical Trials in Early Alzheimer Disease Incorrectly?. JAMA Neurol.

[6] Daly T, Epelbaum S (2023). The Accelerated Approval of Aducanumab Invites a Rethink of the Current Model of Drug Development for Alzheimer's Disease. AJOB Neurosci.

[7] Piller C (2023). Report on trial death stokes Alzheimer's drug fears. Science.

[8] Daly T, Henry V, Bourdenx M (2023). From Association to Intervention: The Alzheimer's Disease-Associated Processes and Targets (ADAPT) Ontology. J Alzheimers Dis.

